# Small-Strain Elastic Properties of EPS: Insights from Video Extensometry

**DOI:** 10.3390/ma19081570

**Published:** 2026-04-14

**Authors:** Kamil Słowiński, Ewa Delalicz de Lawal

**Affiliations:** 1 Faculty of Civil Engineering, Silesian University of Technology in Gliwice, Akademicka 5, 44-100 Gliwice, Poland; 2 Termo Organika Sp. z o.o., Gen Zielińskiego 24, 30-320 Kraków, Poland; edelalicz@termoorganika.pl

**Keywords:** expanded polystyrene (EPS), compression modulus of elasticity, Poisson’s ratio, video extensometer (VE), small-strain behaviour

## Abstract

**Highlights:**

- The modulus of elasticity determined using a video extensometer (VE) was up to twice that from crosshead displacement measurements.
- VE measurements showed no bedding error even at a gauge length of 95% of the specimen height.
- The EPS stress–strain behaviour within the elastic range is nonlinear.
- Poisson’s ratio in the small-strain range is sensitive to specimen geometry.
- The video extensometer enables the accurate determination of both the modulus of elasticity and Poisson’s ratio of EPS.
- A bilinear rather than a linear elastic model is recommended for structural applications of EPS.
- Standard 50 mm cubic specimens underestimate the modulus of elasticity.

**Abstract:**

Accurate determination of the elastic parameters of expanded polystyrene (EPS) in the small-strain range is essential for its structural applications. This study investigates the compression modulus of elasticity and Poisson’s ratio of EPS with a nominal density of 20 kg/m^3^ through unconfined compression tests on cubic specimens of 50, 100, 200 and 300 mm side length and rectangular 50 × 50 × 100 mm specimens. Three deformation measurement methods were compared: video extensometer (VE) and crosshead displacement measurements performed at two independent laboratories. Two moduli of elasticity, *E*_0–0.5_ and *E*_0.5–1.0_, were determined within vertical strain (*ε*_v_) ranges of 0–0.5% and 0.5–1.0%, respectively. VE-based moduli were up to twice the values obtained from crosshead displacement measurements, demonstrating that global deformation measurements significantly underestimate material stiffness in the small-strain range due to bedding error. The stress–strain relationship within *ε*_v_ ≤ 1%, commonly regarded as the elastic range of EPS, was found to be clearly nonlinear and is well described by a bilinear model. No size effect on moduli was observed in VE measurements, while crosshead displacement measurements showed increasing moduli with specimen height, stabilising at 100 mm and above. VE-based Poisson’s ratio *v*_0–0.5_ was 0.23 for cubic specimens and 0.09 for rectangular specimens, suggesting a significant effect of specimen geometry. The results highlight the importance of local strain measurement for accurate characterisation of EPS elastic behaviour, particularly in structural applications.

## 1. Introduction

Expanded polystyrene (EPS) is currently widely used not only as an insulating material but also as a structural one [1]. EPS belongs to the group of rigid closed-cell polymer foams. The presence of air in the closed cells gives this material excellent thermal insulation properties, making it widely used for thermal insulation of building walls and roofs, primarily in external thermal insulation composite systems (ETICS) [2,3]. The use of EPS in these systems represents one of the most effective means of reducing heat loss and thus improving building energy efficiency, although end-of-life disposal and recyclability of EPS remain challenges that require further attention [4,5].

The widespread use of EPS, however, is not limited to its insulating properties. The material is also used in protective helmets and protective packaging, where its ability to absorb impact energy is utilised [6]. This property results from its appropriate stiffness and load-bearing capacity combined with the ability to undergo substantial permanent deformation without evident structural disintegration [7]. These mechanical properties, particularly load-bearing capacity and stiffness, also find broad application in geotechnical engineering [8]. EPS is used there as a lightweight fill in road subbase and railway embankment construction [9,10,11,12,13,14,15,16], as a material reducing earth pressure on underground structures [17], and as lightweight structural backfill [18]. In such applications, EPS carries loads arising from earth pressure under self-weight and service loading.

In recent years, EPS has also found application as a protective element for structures, including buildings [19], bridges [20,21], and retaining walls [22,23], against seismic actions, mining-induced tremors, and dynamic excitations. One of the most widespread structural applications of EPS in construction, however, is its use as the core material in structural insulated panels (SIP), employed as wall and roof elements in buildings. In addition to providing thermal insulation, these panels are capable of carrying self-weight loads and climatic actions such as snow and wind [24,25]. In recent years, driven by increasingly stringent building energy efficiency requirements, solutions have emerged in which EPS simultaneously serves as both an insulating material and a load-bearing element in self-supporting composite partitions of residential buildings [26].

Such structural applications require accurate knowledge of EPS material parameters. One of the most commonly performed tests on this material is the unconfined compression test. Its results, particularly compressive strength and compressive modulus of elasticity, form the basis of quality control carried out by EPS manufacturers. While the literature on compression strength methodology and values is broadly consistent, certain ambiguities arise with regard to methods for determining the modulus of elasticity.

The most commonly used measurement method in uniaxial compression tests on EPS involves recording deformation over the full specimen height [7,27,28,29,30,31,32]. In practice, strains are most often determined from crosshead displacement or using additional displacement transducers. This method is widely used both in research and in quality control by EPS manufacturers [33,34].

The limited number of publications in which EPS specimen deformations were recorded both globally—from crosshead displacement—and locally over a gauge length shorter than the full specimen height indicate notable differences in the stress–strain relationship in the loading direction (*σ*-*ε*_v_) within the small-strain range *ε*_v_ ≤ 1%. In [35], both LVDT transducers recording platen displacement and local deformation transducers (LDTs) were used to measure vertical deformations of EPS specimens of nominal density 20 kg/m^3^ and 30 kg/m^3^. Local measurements covered approximately 80% of the specimen height, excluding the contact zones at the loading platens. Significant differences were found between *σ*-*ε*_v_ curves determined by both measurement methods within *ε*_v_ ≤ 1%. Strains determined from platen displacement were consistently greater than those from local measurements, leading to noticeably lower values of both the initial modulus *E*_0_ and tangent modulus *E*_tan_ calculated from global measurements. The primary cause of this phenomenon is identified as the bedding error at the specimen–platen contact zones, resulting in lower stiffness of the material adjacent to the upper and lower specimen surfaces compared to the central region [27,29,30]. The nature of this phenomenon is described in detail in [30], which identifies the irregular specimen surface arising from open voids between polystyrene beads damaged during specimen cutting as the principal cause.

The bedding error is also identified as the primary cause of the considerable scatter in modulus of elasticity values obtained for specimens of different dimensions. As demonstrated in [28,29,34], decreasing specimen dimensions leads to progressively greater underestimation of the initial modulus of elasticity. Modulus values determined for standard 50 mm cubic specimens were found to be up to twice lower than those obtained for 600 mm specimens [29], with the latter being closer to values observed under field conditions [30].

At the same time, the results indicate that the influence of bedding errors decreases with increasing specimen dimensions. In [29,30], the moduli of elasticity determined for 600 mm cubic specimens from both global measurements and local measurements taken over the central third of the specimen height were found to be comparable. Similarly, the authors of [35] reported that initial modulus of elasticity values determined from local measurements on cylindrical specimens of 75 mm diameter and 100 mm height were comparable to results obtained for large 600 mm cubic specimens.

For this reason, in order to accurately determine the modulus of elasticity from relatively small specimens, such as 50 mm cubic specimens, the use of local deformation measurements is recommended, excluding the specimen–platen contact zones, by means of displacement transducers mounted directly on the specimen surface [7,11]. Mounting such transducers can, however, be problematic, particularly for small specimens, and the potential influence of the mounting method on measurement results may also raise concerns.

Precise recording of EPS specimen deformations can be achieved using a video extensometer (VE). This non-contact optical method determines displacements and strains by tracking discrete surface markers, in contrast to full-field digital image correlation (DIC) techniques based on random speckle patterns [36,37,38]. It is widely used in determining mechanical parameters of structural materials, including metals. Its principal advantages are high measurement accuracy and the absence of physical contact with the specimen, enabling local measurements in regions unaffected by bedding errors. To the best of the authors’ knowledge, only a limited number of publications reporting video extensometer (VE) measurements on EPS specimens are available in the literature.

This paper presents the results of unconfined compression tests on EPS specimens of nominal density 20 kg/m^3^, as used in the insulating and load-bearing core of a self-supporting composite partition [26]. The aim of the study was to accurately determine the modulus of elasticity and Poisson’s ratio, of particular importance in the context of structural EPS applications. The results obtained from crosshead displacement and VE measurements, both performed at the university laboratory, were compared with results from tests performed at the EPS manufacturer’s laboratory on specimens from the same material batch. Tests were conducted on cubic specimens with side lengths of 50, 100, 200, and 300 mm and on rectangular specimens of 50 × 50 × 100 mm. Differences between VE results and measurements performed at two different test setups—the university laboratory and the EPS manufacturer’s laboratory—are discussed. Selected practical aspects of applying the VE measurement method are also addressed.

## 2. Test Programme

### 2.1. Materials and Specimens

Cubic specimens with side lengths of 50, 100, 200, and 300 mm and rectangular specimens with a 50 × 50 mm cross-section and 100 mm height were used in the study. The selection of 50 mm cubic specimens reflects their widespread use in both research and quality control by EPS manufacturers [31,39,40]. Larger cubic specimens were used to assess the effect of specimen dimensions on the modulus of elasticity determined from DIC and crosshead displacement measurements. Rectangular 50 × 50 × 100 mm specimens, recommended among others in [11], were included to assess the effect of specimen shape, i.e., the height-to-base dimension ratio, on the determined mechanical parameters.

**Figure 1:**
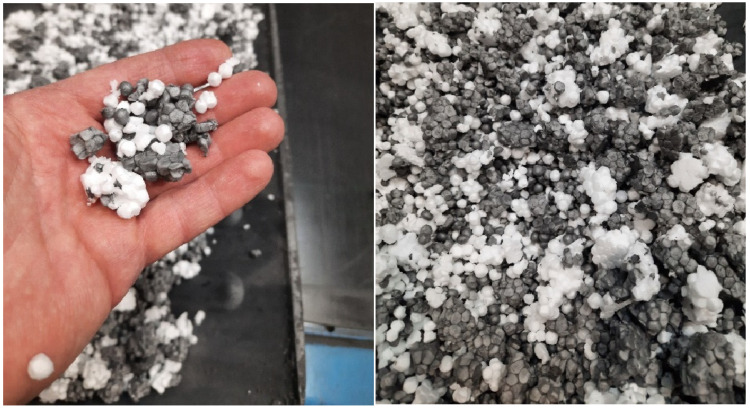
Regrind from mechanical recycling used as an additive in the EPS production process.

All specimens were cut from a single EPS block of nominal bulk density *ρ*_nom_ = 20 kg/m^3^ as declared by the manufacturer. The material also contained approximately 3% regrind from the mechanical recycling of EPS boards. This regrind typically has lower density than the virgin material (approximately 15 kg/m^3^) and occurs as clusters of several to several dozen fused granules (Figure 1).

**Figure 2:**
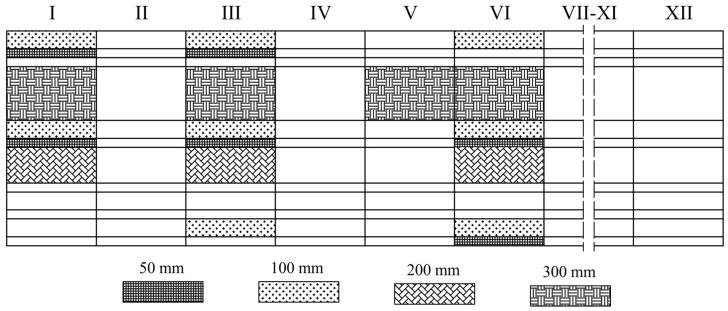
Scheme of EPS block division into sub-blocks (I–XII) and boards of varying thickness.

**Figure 3:**
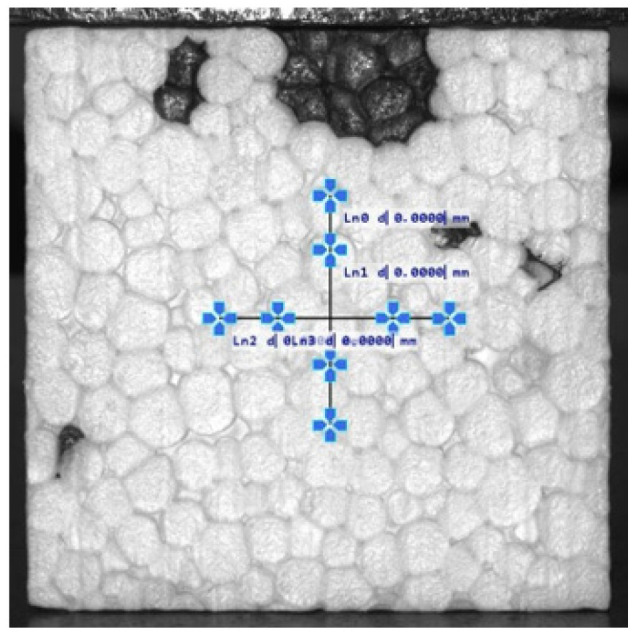
Surface of a 50 mm cubic specimen showing visible regrind clusters (dark areas).

A block of material measuring 1 × 1.2 × 6 m, after 7 days from production, was divided into smaller sub-blocks and subsequently cut into boards of 50, 100, 200, and 300 mm thickness. Figure 2 indicates the boards from which test specimens were taken. Specimens were taken from both the central regions and edge zones of the boards. Specimen cutting was performed using a hot wire cutter. To limit the number of variables, all specimens were prepared at the EPS manufacturer’s facility. Figure 3 shows the surface of a 50 mm cubic specimen, on which darker areas corresponding to regrind clusters are visible.

At the manufacturer’s laboratory, tests were planned for cubic specimens of 50 and 100 mm side length, limited by the load capacity of the available testing machine (Table 1). At the Faculty of Civil Engineering laboratory of the Silesian University of Technology, tests were planned for cubic specimens of all specified dimensions, as well as rectangular specimens (Table 1).

After cutting, the specimens were conditioned for 4 days at a temperature of 23 ± 2 °C and a relative humidity of 50 ± 5%. The apparent density *ρ*_a_ of the specimens was then determined, also at the manufacturer’s facility. The obtained *ρ*_a_ values ranged from 93% to 120% of the nominal material density *ρ*_nom_.

### 2.2. Test Procedure

#### 2.2.1. Tests in the EPS Manufacturer’s Laboratory

**Figure 4:**
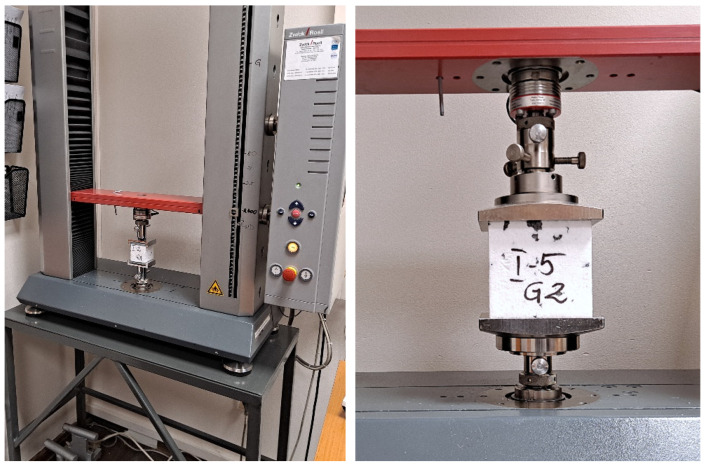
Test setup for compression testing of EPS specimens at the manufacturer’s laboratory.

Tests were performed using a Zwick Roell testing machine, type BT1-FB005TN.D14 (ZwickRoell GmbH & Co. KG, Ulm, Germany) with a maximum load capacity of 2.5 kN (Figure 4). Specimens were loaded monotonically at a strain rate of 10%/min in accordance with [41], until a vertical strain of 60% was reached or the load capacity of the testing machine was exceeded. It should be noted that the strain rate can influence the mechanical properties of viscoelastic materials like EPS. While some studies reported in the literature adopt considerably lower strain rates, such as 0.33%/min or 0.66%/min [35], a rate of 10%/min was adopted in the present study, as it is the standard for quality control testing by EPS manufacturers.

During the tests, the force acting on the specimen and the crosshead displacement were recorded with a resolution of 1/100 N and 1/1000 mm, respectively. Displacement measurement commenced after the application of a preload of approximately 250 Pa, in accordance with [41].

Tests were performed using loading platens designed for EPS testing, connected to the machine crossheads via articulated joints. This test configuration is typical for EPS quality control and also represents the most common approach to compression testing and result recording found in the literature, excluding tests performed in a triaxial apparatus. Furthermore, preliminary tests were conducted without specimens—with the loading platens in direct contact—which confirmed that the machine compliance was negligible relative to the low modulus of the EPS.

#### 2.2.2. Test Procedure at the University Laboratory

**Figure 5:**
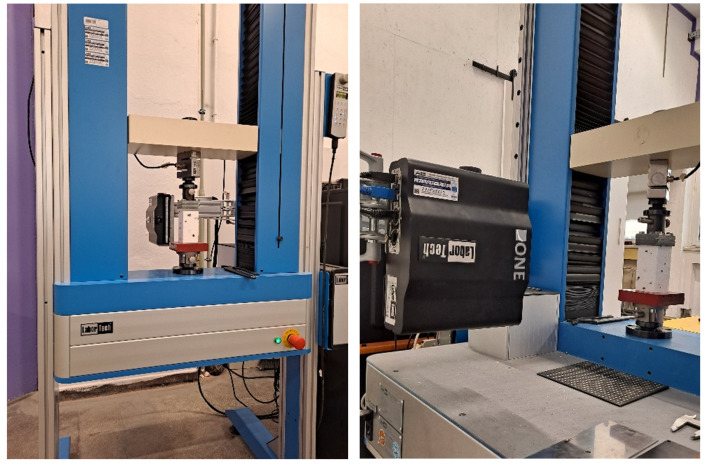
Test setup at the Faculty of Civil Engineering laboratory of the Silesian University of Technology, with the VE camera visible in the foreground (right).

Tests were performed using a Labortech testing machine LabTest 6.50.1.20 (LABORTECH s.r.o., Opava, Czech Republic) with a load capacity of 50 kN and force measurement resolution of 1/100 N (Figure 5). Loading platens made of steel plates of at least 20 mm thickness were used.

Immediately before testing, the density of specimens prepared at the manufacturer’s facility was re-measured. Mass differences did not exceed 0.01 g. The same loading procedure as used in the manufacturer’s laboratory tests was applied. The key difference, however, was the deformation measurement method. Deformations were recorded not only from crosshead displacement but also using a video extensometer (VE) system.

The VE system enabled the measurement of specimen deformation, excluding zones in the immediate vicinity of the upper and lower specimen surfaces, and also reduced the influence of test setup compliance on the results. It was assumed that VE measurements would enable a more precise determination of the deformation of the material itself.

**Figure 6:**
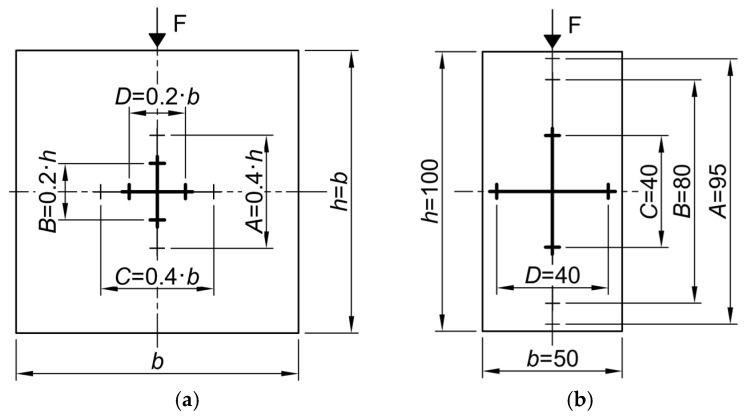
Scheme of video extensometer (VE) gauge lengths on specimen surfaces: (**a**) cubic specimens; (**b**) rectangular specimens (dimensions in mm).

Deformation measurements, both from the testing machine and using the VE system, were recorded with a resolution of 1/1000 mm. A single Labortech xSight One video extensometer (LABORTECH s.r.o., Opava, Czech Republic) with a resolution of 1 μm was used (Figure 5). Measurements were taken on one lateral surface of each specimen. Four deformation gauge lengths were defined on this surface. For cubic specimens, two vertical and two horizontal strain measurements were taken. Gauge lengths A and B were set equal to 40% and 20% of the specimen height (*h*), respectively, while C and D were set equal to 40% and 20% of the specimen width (*b*) (Figure 6a). For rectangular specimens, three vertical gauge lengths (A, B, and C) of 95%, 80%, and 40% of the specimen height (*h*), respectively, and one horizontal gauge length (D) of 80% of the specimen width (*b*) were applied (Figure 6b). The use of varying gauge lengths was intended to assess the potential effect of gauge length on measurement results. Deformation measurement commenced after the application of a preload of approximately 500 Pa.

### 2.3. Method for Determining EPS Elastic Parameters

#### 2.3.1. Elastic Range of EPS Behaviour in the Literature

Descriptions of the elastic range of EPS behaviour available in the literature indicate a varied character of the stress–strain relationship *σ*-*ε*_v_, which substantially contributes to the scatter of reported compression modulus of elasticity values.

Standards [41,42] recommend determining the modulus of elasticity from the slope of the tangent to the linear portion of the *σ*-*ε*_v_ curve below the proportionality limit. In practice, identifying this portion may prove problematic and subjective. Consequently, the modulus value may depend on the assumed extent of the linear range, and moduli determined for specimens from the same material batch may exhibit considerable variation.

The elastic limit of EPS is most commonly associated in the literature with vertical strains of approximately *ε*_v_ = 1% [7,11,43,44] or within the range of 1–2% [28,31,45]. It should be noted, however, that many of these and other studies [46,47] focus on material behaviour at considerably larger deformations, reaching several tens of percent, which does not provide a complete picture of elastic properties in the small-strain range.

More detailed small-strain analyses indicate, however, that EPS behaviour up to approximately 1% strain is clearly nonlinear. In [48], the modulus of elasticity determined within the strain range up to 0.5% was shown to be approximately 130% of the modulus determined within 0.5–1%. In [27], for EPS of density 20 kg/m^3^, the elastic range extended over strains of 0.43–0.53%, while at approximately 1% strain, the material had already entered an elasto-plastic state. Strongly nonlinear EPS behaviour in the small-strain range was also confirmed in [35], where precise LDT measurements demonstrated that the tangent modulus of elasticity remained approximately constant only up to strains of approximately 0.1%, beyond which it decreased significantly.

With regard to Poisson’s ratio, the relevant standards do not specify a detailed determination procedure. Following common convention, it is defined as the ratio of lateral to longitudinal strain *ε*_h_/*ε*_v_, within the range below the material proportionality limit. In the literature, however, data on this parameter appear considerably less frequently than data on compressive strength or the modulus of elasticity. A broader discussion of available literature data is presented in Section 4.6.2.

#### 2.3.2. Procedure for Determining Moduli of Elasticity from Test Results

**Figure 7:**
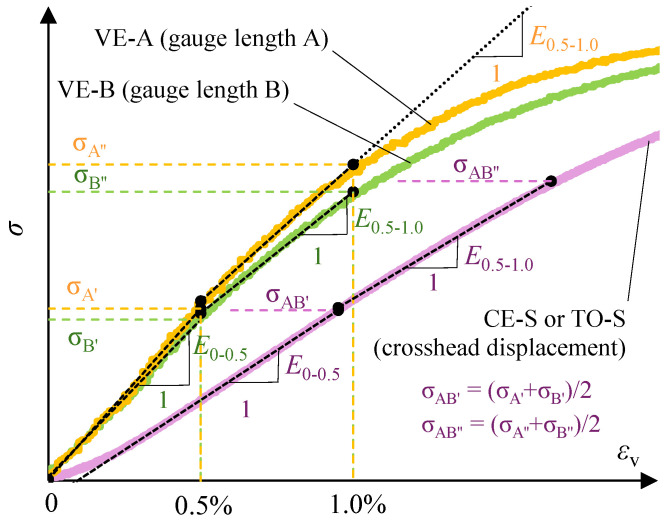
Scheme for determining compression moduli of elasticity *E*_0–0.5_ and *E*_0.5–1.0_.

In the present study, two moduli of elasticity were adopted to describe the elastic range of material behaviour, *E*_0–0.5_ and *E*_0.5–1.0_, characterising material behaviour within vertical strain ranges of 0–0.5% and 0.5–1%, respectively (Figure 7). These ranges were adopted on the basis of observations reported in the literature, indicating clearly the nonlinear character of the *σ*-*ε*_v_ relationship in the small-strain range (see Section 2.3.1). These strain ranges were used directly to determine moduli from *σ*-*ε*_v_ curves obtained from VE measurements.

For curves designated CE-S and TO-S, determined from crosshead displacement measurements at the SUT laboratory and the EPS manufacturer’s laboratory, respectively, the segments used for modulus determination were bounded by stress levels σ_AB’_ and σ_AB’’_ corresponding to the mean values of σ_A’_ and σ_B’_, and σ_A’’_ and σ_B’’_, respectively. These values were read from the VE curves at strain levels *ε*_v_ of 0.5% and 1%, respectively (Figure 7).

VE measurements were taken as the reference for determining moduli of elasticity from crosshead displacement measurements. As noted previously, deformation measurements based on crosshead displacement may be affected by additional error arising from test setup compliance, leading to overestimation of the recorded deformations. Consequently, the strain criterion determined directly from crosshead displacement may be unreliable [35].

Within the result group TO-S, the described modulus determination methodology was applied only to 9 specimens taken from the same boards, in the immediate vicinity of specimens designated for testing at the SUT laboratory. The modulus determination method for the remaining specimens tested at the EPS manufacturer’s laboratory (result group TO-S*) is presented in Section 2.3.3.

#### 2.3.3. Estimation Procedure for Moduli in the Result Group TO-S*

For the 26 specimens tested at the EPS manufacturer’s laboratory that had no counterparts among specimens designated for testing at the SUT laboratory, it was necessary to determine the limiting stresses *σ*_AB’_ and *σ*_AB’’_ (see Figure 7) for calculating moduli *E*_0–0.5_ and *E*_0.5–1.0_.

The compressive strength at 10% strain, *σ*_10_, was adopted as the reference material parameter for determining these values. In numerous studies, *σ*_10_ is treated as largely insensitive to specimen shape and dimensions [28,29,33,34,40], with its value depending primarily on material density. It can therefore be expected that *σ*_10_ for all specimens will exhibit a consistent trend and good correlation with material density, regardless of the measurement method or specimen shape used.

It was assumed that *σ*_AB’_ and *σ*_AB’’_ would be determined from a regression-based relationship between compressive strength *σ*_10_ and moduli *E*_0–0.5_ and *E*_0.5–1.0_, obtained from VE measurements, which were considered to best reflect the true behaviour of the tested EPS material. The resulting values of *σ*_AB’_ and *σ*_AB’’_ define the boundaries of the *σ*-*ε*_v_ curve segments (TO-S* curves) used for calculating moduli *E*_0–0.5_ and *E*_0.5–1.0_ for the considered group of 26 specimens.

Stresses *σ*_AB’_ and *σ*_AB’’_ were determined iteratively. In the first step, an approximate σ_10_ was obtained from the modulus of elasticity calculated as the slope of the tangent to the linear portion of the *σ*-*ε*_v_ curve, following the standard approach recommended in [41]. An approximate σ_AB’_ was then calculated from the VE-based regression relationships and used to determine a preliminary *E*_0–0.5_, from which σ_10_ was recalculated. The σ_10_ thus obtained was used to determine the final values of σ_AB’_ and σ_AB’’_, applied to calculate *E*_0–0.5_ and *E*_0.5–1.0_ from the experimental curves TO-S*.

#### 2.3.4. Procedure for Determining Poisson’s Ratios

VE measurements of strains both in the loading direction and perpendicular to it enabled the determination of Poisson’s ratio for the tested material. Two Poisson’s ratios were adopted to describe the elastic range, *v*_0–0.5_ and *v*_0.5–1.0_, corresponding to vertical strain ranges *ε*_v_ of 0–0.5% and 0.5–1.0%, respectively.

**Figure 8:**
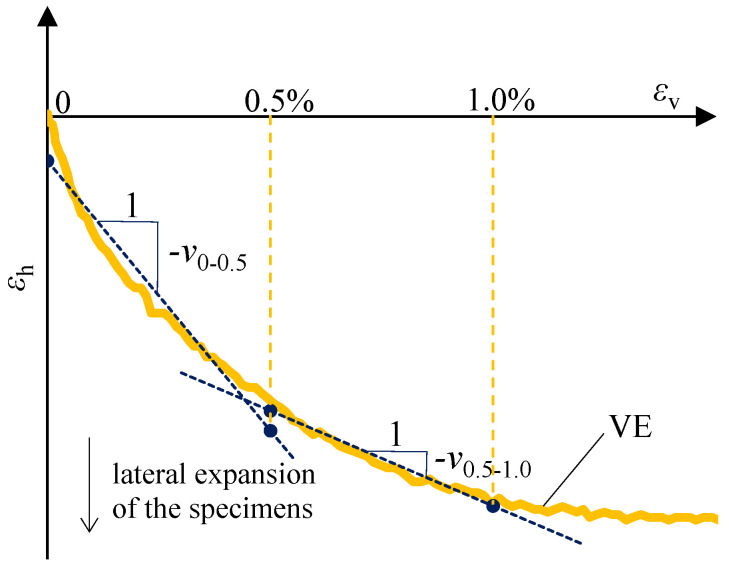
Scheme for determining Poisson’s ratios *v*_0–0.5_ and *v*_0.5–1.0_.

Poisson’s ratios were determined following the scheme shown in Figure 8, from the relationship between horizontal strain *ε*_h_ and vertical strain *ε*_v_ obtained from VE measurements, as the negatives of the slopes of regression lines fitted to the corresponding segments of the *ε*_h_-*ε*_v_ relationship.

The *ε*_h_-*ε*_v_ relationships were established from measurements taken on gauge lengths C and A or D and B for cubic specimens, and D and C for rectangular specimens (Figure 6).

## 3. Results

### 3.1. Overview of Test Results

**Figure 9:**
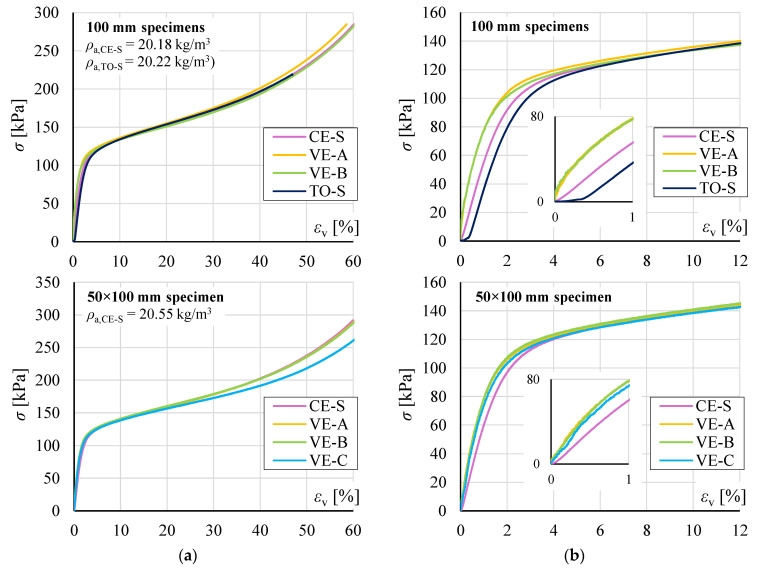
Representative stress *σ* versus vertical strain *ε*_v_ relationships: (**a**) full range up to 60% strain; (**b**) enlarged view up to 12% strain with inset showing the elastic range up to 1% strain.

Figure 9 presents representative stress *σ* versus vertical strain *ε*_v_ relationships for cubic and rectangular specimens. Results from the Faculty of Civil Engineering laboratory are designated CE-S, VE-A, VE-B, and VE-C, while results from the EPS manufacturer’s laboratory are designated TO-S.

Curves CE-S and TO-S were determined from crosshead displacement, while curves VE-A, VE-B, and VE-C correspond to VE measurements for gauge lengths A, B, and C (Figure 6). The comparison of CE-S and TO-S curves is based on results from specimens cut from directly adjacent regions of the EPS boards, with similar apparent density.

A characteristic feature of TO-S curves was a pronounced region of large initial deformations, often visible as a rounded, slightly concave segment preceding the linear portion of the relationship (Figure 9b). In CE-S curves, this region was considerably less prominent, while in DIC results, it was not observed. In all cases, VE curves exhibited greater stiffness in the quasi-linear strain range than CE-S and TO-S curves.

The *σ*-*ε_v_* curves for all specimens showed the characteristic phases of EPS behaviour: an initial quasi-linear response at small strains *ε*_v_ (up to a few percent), followed by a phase of intensive deformation growth, and a final densification phase accompanied by renewed stiffness increase (Figure 9a).

**Figure 10:**
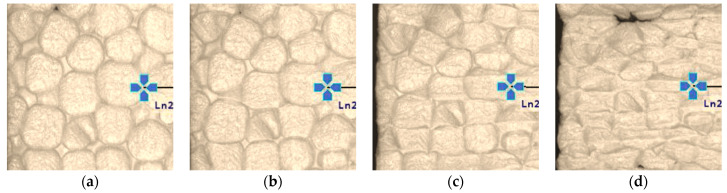
Changes in EPS structure at strain levels *ε*_v_ of: (**a**) 0%; (**b**) 10%; (**c**) 30%; and (**d**) 60%.

Figure 10 presents visually observable changes in material structure accompanying load increase. Within the quasi-elastic strain range (*ε_v_* ≤ 1%), the primary observation was a reduction in inter-bead spacing (Figure 10a). At larger strains, local shape changes appeared at bead contact points, initially accompanied by slight surface wrinkling in the contact zones (Figure 10b,c). For *ε*_v_ > 10%, extensive and pronounced surface wrinkling and further bead deformation were observed (Figure 10c,d). Neither during testing nor after unloading were any cracks or other forms of brittle damage observed that could indicate loss of structural integrity, consistent with findings reported in the literature [49].

**Figure 11:**
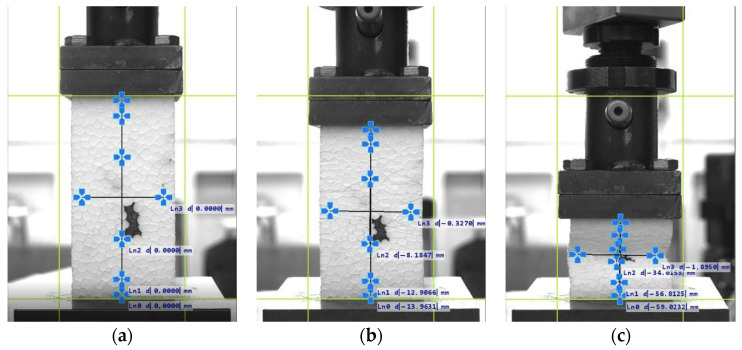
Behaviour of 50 × 100 mm specimens at strain levels *ε*_v_ of: (**a**) 0%; (**b**) 30%; and (**c**) 60%, showing visible bending deformations.

For all 50 × 100 mm specimens, significant bending deformations were observed during testing (Figure 11). These became visible to the naked eye at strains *ε*_v_ (from crosshead measurement) exceeding approximately 5%. Consequently, *ε*_v_ measurements, particularly at large deformations, may be affected by error due to the bending moment. Additionally, these deformations caused changes in the distance between the VE camera and the specimen surface, potentially introducing further measurement error.

Comparison of curves obtained for 50 × 100 mm specimens with those for 100 mm cubic specimens of similar density revealed no significant differences within *ε*_v_ ≤ 10%. In particular, no bending moment effect was observed in the small-strain range *ε*_v_ ≤ 1%. The results for rectangular specimens were therefore considered suitable for determining both moduli of elasticity and compressive strength at 10% strain.

**Figure 12:**
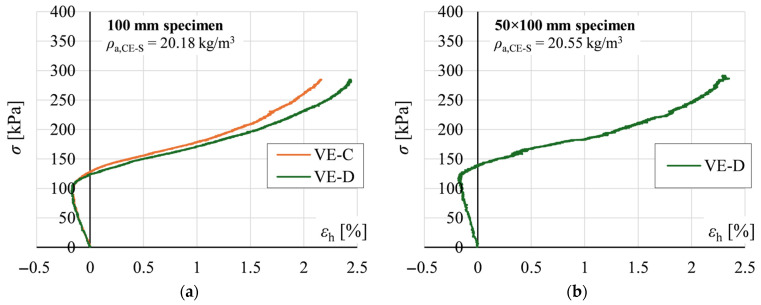
Representative stress *σ* versus horizontal strain *ε*_h_ relationships for: (**a**) 100 mm cubic specimen; and (**b**) 50 × 100 rectangular specimen.

**Figure 13:**
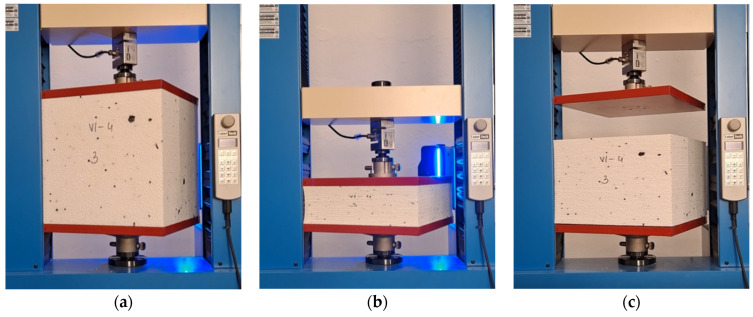
Deformations of a 300 mm cubic specimen: (**a**) after preload application; (**b**) at strain *ε*_v_ = 60%; and (**c**) after unloading.

Representative stress *σ* versus horizontal strain *ε*_h_ relationships are shown in Figure 12. Curves VE-C and VE-D for cubic specimens, and VE-D for rectangular specimens, were determined using the gauge lengths defined in Figure 6. In the initial loading phase, increasing horizontal strains indicated lateral expansion of the specimens. At stress levels corresponding to the elasto-plastic range of the *σ*-*ε*_v_ curves, the direction of *ε*_h_ reversed, indicating lateral contraction. This phenomenon was particularly pronounced for 200 and 300 mm cubic specimens, which developed a clear hourglass shape in the final loading phase (Figure 13).

Changes in the distance between the video extensometer and the specimen surface, accompanying lateral dimension changes, may have introduced additional measurement error. However, as observed, horizontal strains *ε*_h_ within the initial loading phase *ε*_v_ ≤ 1% generally did not exceed 0.25%. The potential measurement error arising from this effect was therefore considered negligible for the determined moduli of elasticity and Poisson’s ratios.

To compare VE results obtained using gauge lengths of different lengths, Table 2 presents stress values corresponding to strains *ε*_v_ of 0.5% and 1%, determined from different gauge lengths. Stresses *σ*_A’_, *σ*_B’_, *σ*_C’_ and *σ*_A’’_, *σ*_B’’_, *σ*_C’’_ were determined from strain measurements using gauge lengths A and B for cubic specimens, and A, B and C for rectangular specimens (Figure 6). The coefficient of variation *CV*, defined as the ratio of standard deviation *s* to the mean, is also given for mean stress ratios.

Similarly, Table 3 compares horizontal strains *ε*_C_ and *ε*_D_ recorded for cubic specimens using gauge lengths C and D (Figure 6a) at strain levels *ε*_v_ of 0.5% and 1%. For specimens of 100 mm height and above, very good agreement was obtained across all gauge lengths. The greatest scatter was observed for 50 mm cubic specimens, though even here, *CV* values indicate only moderate variability.

### 3.2. Compression Moduli of Elasticity Determined from Crosshead Displacement Measurements

**Figure 14:**
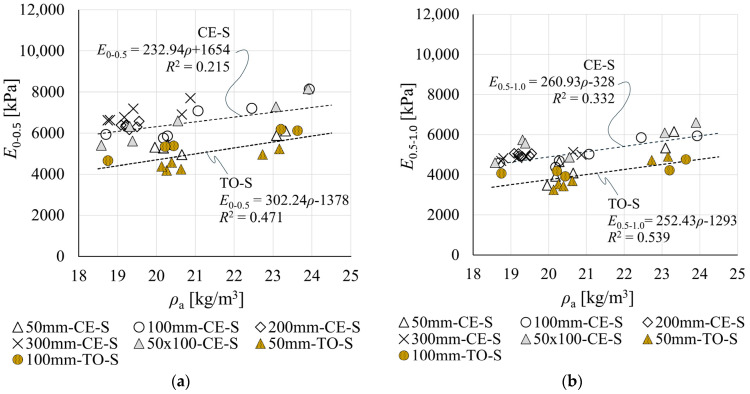
Compression moduli of elasticity determined from testing machine measurements as a function of EPS apparent density *ρ*_a_ for: (**a**) modulus *E*_0–0.5_; and (**b**) modulus *E*_0.5–1.0_.

Figure 14 presents moduli of elasticity *E*_0–0.5_ and *E*_0.5–1.0_ determined from crosshead displacement measurements at the SUT laboratory (CE-S) and at the EPS manufacturer’s laboratory (TO-S), as a function of apparent density *ρ*_a_. The strong influence of material density on EPS mechanical parameters is well established in the literature. Although all specimens shared the same nominal density *ρ*_nom_, the measured *ρ*_a_ ranged from 93% to 120% of the *ρ*_nom_ value.

In both result groups (CE-S and TO-S), moduli *E*_0–0.5_ and *E*_0.5–1.0_ increase with increasing *ρ*_a_. The low coefficients of determination *R*^2^ indicate a relatively weak correlation between the measurements and the fitted regression lines, though it should be noted that the density range analysed is relatively narrow.

The lowest moduli were obtained for the smallest specimens—50 mm cubic—in both CE-S and TO-S groups. In group CE-S, these results also exhibit the greatest scatter and deviate markedly from the trend established by larger specimens. The results for 50 × 100 mm rectangular specimens, by contrast, fit this trend satisfactorily.

Moduli obtained in group CE-S are higher than in group TO-S. Regression-based *E*_0–0.5_ values for density *ρ*_a_ in the range 18.5–24 kg/m^3^ are 5963–7245 kPa for CE-S and 4213–5876 kPa for TO-S. The corresponding *E*_0.5–1.0_ ranges are 4499–5934 kPa and 3377–4765 kPa, respectively.

At nominal density *ρ*_nom_ = 20 kg/m^3^, regression-based *E*_0–0.5_ and *E*_0.5–1.0_ for CE-S are 135% and 130% of the corresponding TO-S values, respectively. Within group CE-S, *E*_0–0.5_ is 129% of *E*_0.5–1.0_, while the corresponding ratio for TO-S is 124%.

### 3.3. Compression Moduli of Elasticity Determined from Video Extensometer Measurements

**Figure 15:**
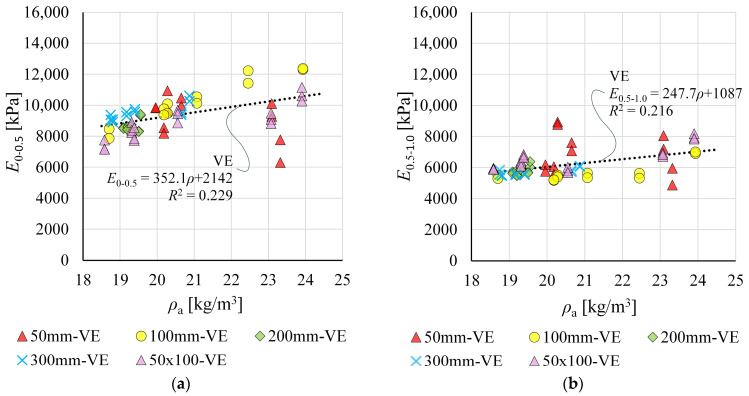
Compression moduli of elasticity determined from VE measurements as a function of apparent density *ρ*_a_ for: (**a**) modulus *E*_0–0.5_; and (**b**) modulus *E*_0.5–1.0_.

Moduli of elasticity *E*_0–0.5_ and *E*_0.5–1.0_ determined from VE measurements are presented in Figure 15. For cubic and rectangular specimens, two and three data points at a given *ρ*_a_ are shown, respectively, corresponding to different gauge lengths.

The results for all specimens follow a trend indicating dependence of *E*_0–0.5_ and *E*_0.5–1.0_ on apparent density *ρ*_a_. The low *R*^2^ values can be attributed to the relatively large scatter observed for 50 mm cubic specimens and partly to the narrow density range analysed.

Regression-based *E*_0–0.5_ and *E*_0.5–1.0_ for density *ρ*_a_ in the range 18.5–24 kg/m^3^ are 8655–10,592 kPa and 5670–7032 kPa, respectively. At nominal density, *E*_0–0.5_ is 152% of *E*_0.5–1.0_.

### 3.4. Estimated Compression Moduli of Elasticity for Result Group TO-S*

Moduli *E*_0–0.5_ and *E*_0.5–1.0_ were determined for 26 of the 35 specimens tested at the EPS manufacturer’s laboratory that had no counterparts among specimens tested at the SUT laboratory (see Section 2.1), following the estimation procedure described in Section 2.3.3.

**Figure 16:**
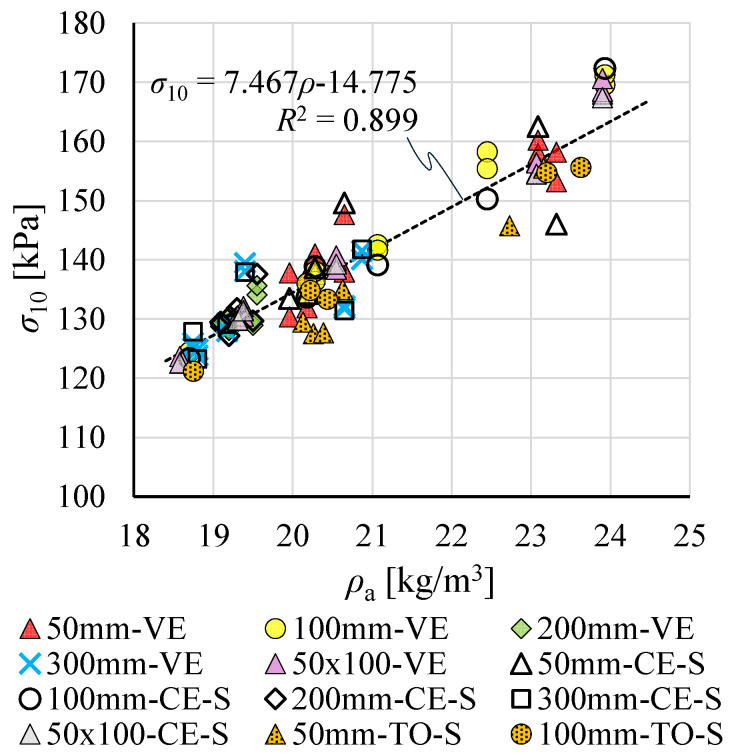
Compressive strength *σ*_10_ as a function of apparent density *ρ*_a_.

The starting point for determining limiting stresses σ_AB’_ and σ_AB’’_ was the observation that compressive strength at 10% strain, *σ*_10_, showed good correlation with apparent density *ρ*_a_, regardless of measurement method, specimen dimensions, or shape (Figure 16).

**Figure 17:**
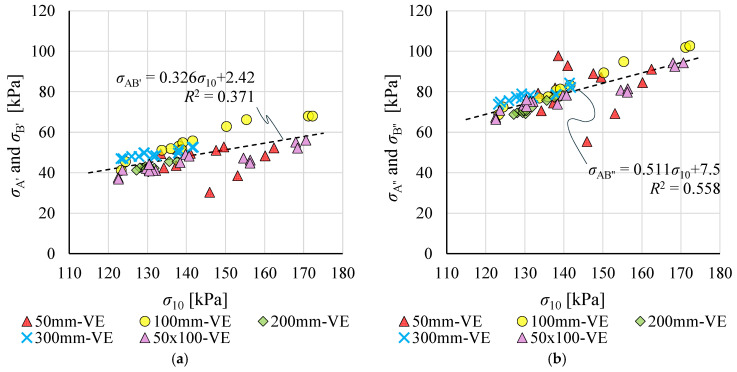
Relationship between compressive strength σ_10_ and stress values used for determining: (**a**) modulus *E*_0–0.5_; and (**b**) modulus *E*_0.5–1.0_.

On this basis, relationships between stresses σ_A’_, σ_B’_ and σ_A’’_, σ_B’’_, and strength σ_10_ were established for the VE result population and approximated by linear functions (Figure 17). These relationships were then used to calculate *σ*_AB’_ and *σ*_AB’’_ for each of the 26 specimens, following the scheme shown in Figure 7.

**Figure 18:**
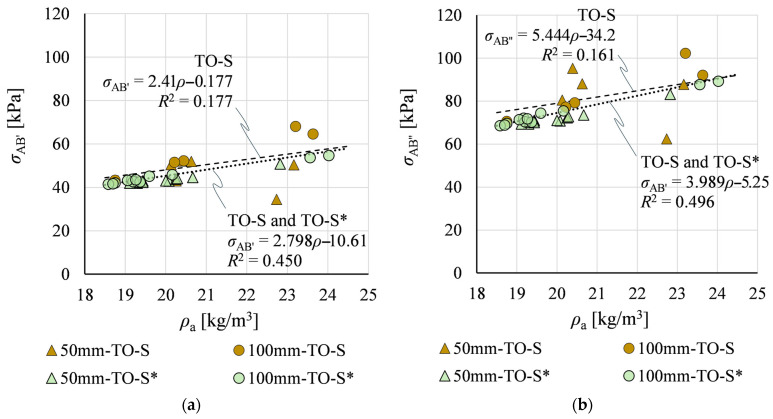
Relationships between stresses *σ*_AB’_ and *σ*_AB’’_ and apparent density *ρ*_a_ for: (**a**) measured (TO-S) data; and (**b**) estimated (TO-S*) data.

Figure 18 compares the resulting *σ*_AB’_ and *σ*_AB’’_—constituting the TO-S* dataset—with stress values previously determined for the nine specimens from group TO-S. The estimated TO-S* results follow the general trends established by TO-S measurements, and *R*^2^ values for regression lines fitted to the combined dataset (TO-S and TO-S*) are noticeably higher than for TO-S data alone.

**Figure 19:**
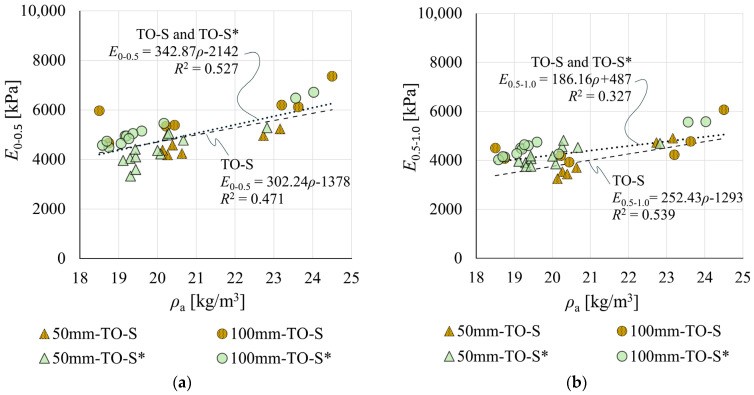
Relationships between moduli *E*_0–0.5_ and *E*_0.5–1.0_ and apparent density *ρ*_a_ for: (**a**) measured (TO-S) data; and (**b**) estimated (TO-S*) data.

Relationships between moduli *E*_0–0.5_ and *E*_0.5–1.0_ and density *ρ*_a_ for TO-S and TO-S/TO-S* data are shown in Figure 19. The difference between regression-based *E*_0–0.5_ at *ρ*_a_ = 20 kg/m^3^ for both datasets did not exceed 1%. The regression-based *E*_0.5–1.0_ for TO-S/TO-S* was 112% of the TO-S value. Despite these differences, the estimated TO-S* results can be considered consistent with the general trend established by TO-S measurements.

### 3.5. Comparison of Compression Moduli of Elasticity Obtained by Different Measurement Methods

**Figure 20:**
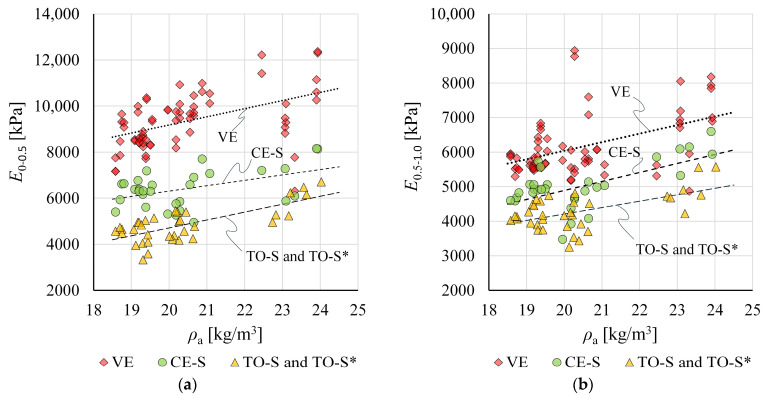
Compression moduli of elasticity as a function of apparent density for all result groups for: (**a**) modulus *E*_0–0.5_; and (**b**) modulus *E*_0.5–1.0_.

Figure 20 presents all determined moduli of elasticity *E*_0–0.5_ and *E*_0.5–1.0_ as a function of apparent density *ρ*_a_. The fitted regression lines follow a similar trend, indicating increasing moduli with increasing *ρ*_a_.

**Figure 21:**
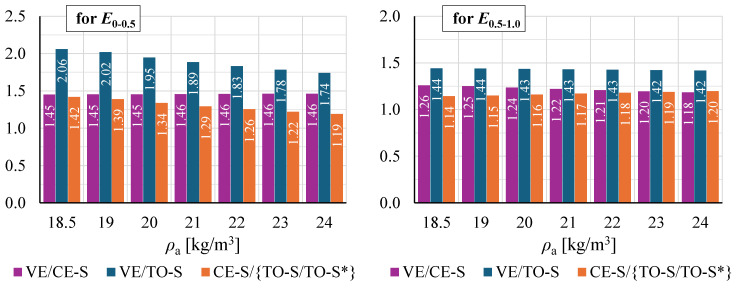
Comparison of moduli of elasticity *E*_0–0.5_ and *E*_0.5–1.0_ determined from regression line equations.

Regression-based moduli for all result groups over the considered density range are compared in Figure 21. Due to differences in regression line slopes, the differences between groups vary across the density range, generally reaching their largest values at the lower end.

VE-based moduli are clearly higher than those from groups CE-S and TO-S/TO-S*, determined from crosshead displacement measurements at the SUT laboratory and the EPS manufacturer’s facility, respectively. The regression-based *E*_0–0.5_ at *ρ*_a_ = *ρ*_nom_ = 20 kg/m^3^ from VE measurements is 195% of the TO-S/TO-S* value and 145% of the CE-S value. Somewhat lower ratios of 143% and 124% were obtained for *E*_0.5–1.0_.

At *ρ*_nom_, moduli *E*_0–0.5_ and *E*_0.5–1.0_ for CE-S are 134% and 116% of the corresponding TO-S/TO-S* values.

### 3.6. Poisson’s Ratios Determined from VE Measurements

**Figure 22:**
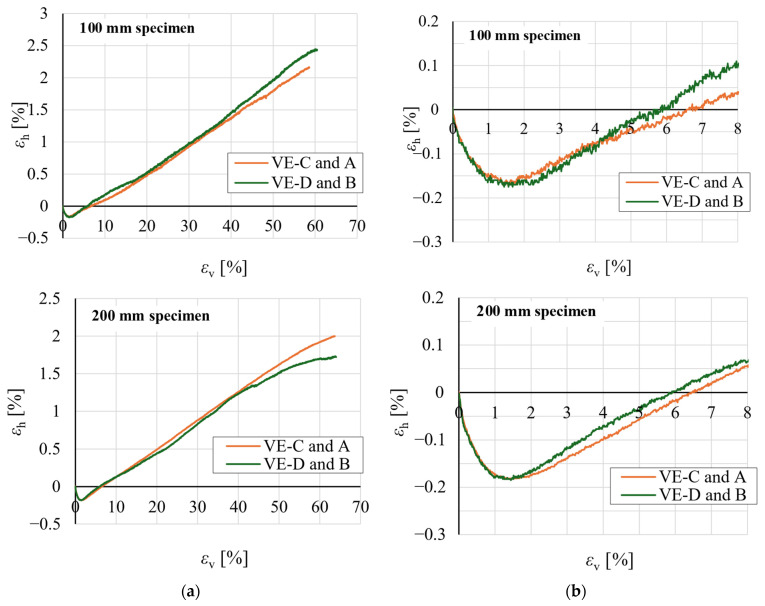
Representative relationships between horizontal strain *ε*_h_ and vertical strain *ε*_v_ for 100 mm and 200 mm cubic specimens: (**a**) full strain range; (**b**) magnified view of the initial strain range.

Figure 22 presents representative relationships between horizontal strain *ε*_h_ and vertical strain *ε*_v_ obtained for cubic specimens. Within the small-strain range corresponding to the elastic behaviour of EPS, horizontal strains were negative, indicating lateral expansion of the specimens (Figure 12). With increasing load, *ε*_h_ became positive, indicating lateral contraction.

**Figure 23:**
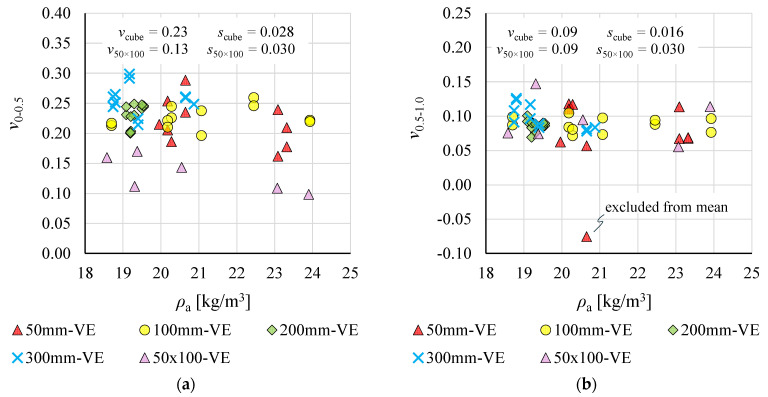
Poisson’s ratios as a function of EPS apparent density for: (**a**) ratio *v*_0–0.5_; and (**b**) ratio *v*_0.5–1.0_.

Poisson’s ratios *v*_0–0.5_ and *v*_0.5–1.0_, determined within strain ranges *ε*_v_ ≤ 0.5% and 0.5 < *ε*_v_ ≤ 1%, respectively, are plotted as a function of apparent density *ρ*_a_ in Figure 23. The results indicate that Poisson’s ratio remains approximately constant over the density range considered.

Poisson’s ratios *v*_0–0.5_ determined for cubic specimens were noticeably higher than those for rectangular specimens. The mean values of *v*_0–0.5_ and *v*_0.5–1.0_ were 0.23 and 0.09 for cubic specimens, and 0.13 and 0.09 for rectangular specimens, respectively. For cubic specimens, the mean *v*_0–0.5_ was 259% of *v*_0.5–1.0_. The corresponding ratio for 50 × 100 mm specimens was 141%.

Notable are the scatter values of Poisson’s ratio, expressed as standard deviations *s*_cube_ and *s*_50 × 100_ for cubic and rectangular specimens, respectively. For *v*_0–0.5_ and *v*_0.5–1.0,_ the standard deviation for cubic specimens amounted to 12% and 18% of the respective mean values *v*_cube_, indicating acceptable result consistency. The corresponding values of *s*_50 × 100_ for rectangular specimens were 23% and 32% of the respective means *v*_50 × 100_, indicating moderate and high statistical variability, respectively.

The greatest scatter was observed for 50 mm cubic specimens. In one case, a negative value of *v*_0.5–1.0_ was recorded; this was identified as an outlier by Grubbs’ test and excluded from the mean calculation for cubic specimens.

## 4. Discussion

### 4.1. Effect of Measurement Method on Compression Modulus of Elasticity and Poisson’s Ratio Values

Moduli of elasticity *E*_0–0.5_ and *E*_0.5–1.0_ determined from video extensometer (VE) measurements were markedly higher than those obtained from crosshead displacement (result groups CE-S and TO-S/TO-S*).

The largest differences were found for *E*_0–0.5_, where the VE-based modulus was up to twice the value obtained in group TO-S/TO-S*, i.e., determined from crosshead displacement in the EPS manufacturer’s tests. It should be noted, however, that this comparison reflects both the effect of the strain measurement method and potential differences in test setup configuration between the SUT laboratory and the EPS manufacturer’s facility.

For assessing the effect of the measurement method alone, the most relevant comparison is between groups VE and CE-S, obtained using the same test setup and specimens. In this case, *E*_0–0.5_ and *E*_0.5–1.0_ determined from VE measurements were approximately 145% and 118–126% of the values obtained in group CE-S, respectively. This indicates that VE measurements captured noticeably higher material stiffness in the small-strain range compared to crosshead displacement-based measurements.

The differences between *E*_0–0.5_ and *E*_0.5–1.0_ values in groups CE-S and TO-S/TO-S* also require comment. Moduli obtained in group CE-S were 134% and 116% of those from group TO-S/TO-S*, respectively. As the specimens were taken from the same material batch and prepared at the EPS manufacturer’s laboratory, following the same test procedure, these differences should be attributed to differing compliance of the test setups at the SUT laboratory and the EPS manufacturer’s facility. It is likely that these differences resulted, among other factors, from variations in the stiffness of the connections between the loading platens and the machine actuators at the two testing setups. VE measurements largely eliminate the influence of such factors, enabling direct observation of the true material response.

Although the results did not show a significant impact of the measurement method on the Poisson’s ratio values, it is worth noting that VE measurements may potentially exhibit higher sensitivity to rate-dependent effects compared to global crosshead displacement. Currently, there is a lack of specific literature data regarding this influence on VE-derived elastic moduli and Poisson’s ratios within the investigated strain rate range. This issue remains a potential subject for future research.

It should be noted that measurements were performed using a single VE camera on one specimen surface only. The good repeatability of the results obtained suggests, however, that this limitation did not significantly affect the findings. The use of two cameras on opposite specimen surfaces with averaged readings is nevertheless recommended for future studies.

### 4.2. Effect of Specimen Dimensions on Modulus of Elasticity Values

No clear effect of specimen dimensions on moduli of elasticity was observed within the VE result group. This effect was, however, apparent to some extent in groups CE-S and TO-S/TO-S*.

The widely reported size effect on the initial modulus of elasticity consists of increasing modulus values with increasing specimen dimensions. The primary cause identified in the literature is the so-called bedding error, arising from non-uniform contact between the specimen end surfaces and the loading platens. As a result, measurements taken over the full specimen height capture excessive deformations occurring in the vicinity of the platens. This error increases with decreasing specimen size, which explains the lowest modulus values obtained for 50 mm cubic specimens.

Differences between results for 50 mm specimens and larger specimens are particularly pronounced for *E*_0–0.5_. Similar findings were reported in [29], where modulus values for 600 mm specimens were approximately twice those obtained for 50 mm specimens. Accordingly, specimens larger than 50 mm are recommended for determining EPS modulus of elasticity [28]. It is worth noting that 50 mm cubic specimens are standard in EPS quality control procedures.

Given the small differences between CE-S results for specimens of 100 mm height and larger, the bedding error may be considered negligible for these specimen sizes.

### 4.3. Effect of Specimen Shape on Material Parameters

No significant differences in modulus of elasticity values were observed between cubic and rectangular specimens in either the VE or CE-S result groups.

The satisfactory agreement between CE-S results for 50 × 100 mm rectangular specimens and cubic specimens of 100 mm height and larger indicates that specimen height, rather than cross-sectional dimensions, is the governing factor for measurement accuracy. As specimen height increases, the contribution of the deformed material layer adjacent to the loading platens to the total specimen deformation diminishes. For CE-S specimens, this effect was negligible for heights of 100 mm and above.

In earlier studies reported in [29,30], the bedding error was found to diminish only for specimens with side dimensions of approximately 600 mm. The significant role of specimen height is further supported by results presented in [40], where increasing modulus of elasticity values with increasing height were demonstrated for cylindrical specimens of constant diameter.

The effect of specimen shape was, however, clearly apparent for Poisson’s ratio *v*_0–0.5_, determined within the strain range *ε*_v_ ≤ 0.5%. The mean value for 50 × 100 mm rectangular specimens was approximately 57% of that obtained for cubic specimens. No such effect was observed for *v*_0.5–1.0_, determined within 0.5 < *ε*_v_ ≤ 1%. No clear explanation for these differences was found in the available literature. Their identification would most likely require additional experimental studies on specimens of varying geometry, as also suggested by the relatively high coefficients of variation *CV* obtained for Poisson’s ratios determined for 50 × 100 mm rectangular specimens.

### 4.4. Effect of EPS Structural Non-Uniformities and VE Gauge Length on Measurement Results

Within the VE result group, both *E*_0–0.5_ and *E*_0.5–1.0_ values for the vast majority of specimens followed a clear trend. An exception was observed for a subset of 50 mm cubic specimens, which exhibited the greatest scatter.

One possible cause is a non-uniform density distribution within these specimens. The influence of local structural irregularities increases with decreasing specimen size, as also indicated by data reported in [29,50]. A second identified cause was the presence of regrind clusters within the specimen material. The regrind consisted of agglomerates of several dozen fused beads, incorporated into the virgin material during EPS block production. As the regrind typically had lower density than the virgin material, its presence caused local structural discontinuities, leading to locally variable mechanical properties.

This is supported by the results presented in Table 2 and Table 3, where the highest coefficients of variation *CV* were obtained for 50 mm cubic specimens across all gauge lengths considered. These differences indicate significant local stiffness variability within these specimens. The results also suggest that for the EPS studied, gauge lengths of 10 mm and 20 mm may not fully capture the representative material properties of the specimens.

For cubic specimens of 100, 200, and 300 mm and rectangular 50 × 100 mm specimens, good agreement was obtained across all gauge lengths, indicating no significant influence of local material non-uniformities on the determined moduli. It is worth noting that similarly large regrind clusters were also observed on the surface of some of these specimens. This confirms that the larger the specimen dimensions and the longer the gauge length, the smaller the influence of such local structural non-uniformities on measurement results.

The results obtained for 100 mm rectangular specimens further indicate that no bedding error effect was apparent even at a gauge length of 95% of the specimen height.

It should be noted that the present study focused on gauge-length-based strain measurements rather than full-field DIC analysis. Full-field strain maps, which could provide additional insight into the spatial distribution of strains, are identified as a direction for future research.

### 4.5. Nature of the Stress–Strain Relationship in the Elastic Range of EPS

Differences between *E*_0–0.5_ and *E*_0.5–1.0_ were observed in all result groups. The largest differences were found for VE-based moduli, where regression-based *E*_0–0.5_ was 151–153% of *E*_0.5–1.0_. The corresponding values in groups CE-S and TO-S/TO-S* were 122–133% and 123–125%, respectively. These differences indicate nonlinear stress–strain behaviour within the strain range *ε*_v_ ≤ 1%, commonly regarded in the literature as the elastic limit of EPS. This is consistent with earlier literature findings (see Section 2.3.1).

VE measurements also capture the nonlinear character of the stress–strain relationship within *ε*_v_ ≤ 1% more effectively than crosshead displacement-based measurements. In the latter case (groups CE-S, TO-S, and TO-S*), the excessive deformations occurring at the specimen–platen contact zones lead to a notable underestimation of *E*_0–0.5_, rendering the *σ*-*ε*_v_ relationship approximately linear within *ε*_v_ ≤ 1%. This explains why the assumption of linear elastic EPS behaviour within this strain range was widely adopted in the earlier literature.

The nonlinear character of deformation within the elastic range is further evidenced by the differences between Poisson’s ratios determined within *ε*_v_ ≤ 0.5% and 0.5% < *ε*_v_ ≤ 1%. For cubic specimens, *v*_0–0.5_ was nearly two and a half times *v*_0.5–1.0_. Strongly nonlinear variation in Poisson’s ratio for EPS, including material of nominal density 20 kg/m^3^, within the above strain ranges was also reported in [35].

In light of the VE results, the description of EPS elastic behaviour may require a more advanced model than a linear one—for example, a bilinear model as adopted in the present study—both for the modulus of elasticity and Poisson’s ratio.

### 4.6. Comparison with Literature Data

#### 4.6.1. Comparison of Moduli of Elasticity

Moduli of elasticity *E*_0–0.5_ and *E*_0.5–1.0_ determined from VE measurements for EPS of nominal density 20 kg/m^3^ are 9184 kPa and 6041 kPa, respectively. The corresponding values obtained from crosshead displacement measurements (CE-S) are 6313 kPa and 4891 kPa, respectively.

Any comparison of these values with moduli reported in the literature can only be indicative. A meaningful comparison would require detailed knowledge of the influence of numerous factors on test results. The scatter observed in the literature reflects, among other things, differences in specimen geometry, loading rate, material density, and structure, including possible regrind addition, as well as different test methods, strain measurement techniques, and criteria used to determine modulus values. Systematic studies fully characterising the influence of each of these factors are currently lacking.

Given the strong dependence of modulus of elasticity on EPS density, the comparison is limited to specimens of nominal density 20 kg/m^3^, consistent with the present study. Initial modulus of elasticity values reported in [7,11,24,27,28,32,39,43,45] range from approximately 4.1 MPa to 7.6 MPa, with strain measurements taken over the full specimen height. These values correspond to moduli obtained in group CE-S and to *E*_0.5–1.0_ from the VE group. The VE-based *E*_0–0.5_, however, is noticeably higher than the values reported in the cited publications.

A higher modulus value of 11.5 MPa, determined from an analytical relationship, was reported in [48], although it was derived from bending tests. It was also shown that the analytical relationship used correlated well with results from 600 mm cubic specimen tests reported in [29]. The lower *E*_0–0.5_ values obtained in the present study may be partly attributed to the presence of regrind. As demonstrated in [7,11], increasing regrind content leads to a reduction in the modulus of elasticity.

A relatively high initial modulus of elasticity of approximately 8.1 MPa—comparable to the VE-based *E*_0–0.5_—was also reported in [35], where strain measurements were taken over 80% of the specimen height, excluding the contact zones at the loading platens and thus limiting the effect of bedding error.

#### 4.6.2. Comparison of Poisson’s Ratio Values

As with the modulus of elasticity, a wide range of Poisson’s ratio values for EPS is reported in the literature. Here too, comprehensive studies identifying correlations between individual factors and Poisson’s ratio are lacking. For this reason, the comparison draws on results from earlier studies conducted on specimens of varying density and geometry, obtained using different test methods.

Poisson’s ratios *v*_0–0.5_ and *v*_0.5–1.0_, obtained from VE measurements, were 0.23 and 0.09 for cubic specimens, and 0.13 and 0.09 for rectangular specimens, respectively. The values obtained for rectangular specimens and *v*_0.5–1.0_ from VE measurements are consistent with values most commonly reported in the literature [7,27,28,51], which generally do not exceed 0.12. Higher values of 0.15 [34] and 0.17 [35] are indicated in a limited number of publications. In this context, the notably higher *v*_0–0.5_ from VE measurements on cubic specimens is of interest. Comparable values are reported in [32,43], where Poisson’s ratio for EPS specimens of density *ρ*_a_ = 20 kg/m^3^ is given as 0.3 and 0.235, respectively.

In [35], Poisson’s ratio values of 0.12 and 0.17 were reported for EPS of density 19.3 kg/m^3^ and 28.0 kg/m^3^, respectively, indicating a dependence between Poisson’s ratio and material density. Such dependence is also implied by analytical relationships presented in [11,18], from which *v* = 0.11 is derived. The results of the present study indicate, however, that *v*_0–0.5_ and *v*_0.5–1.0_ were independent of material density. It should be noted, however, that the density range analysed was relatively narrow, covering specimens of the same nominal density. The wide scatter of Poisson’s ratio values reported in the literature highlights the need for further research in this area, particularly in the context of structural EPS applications where accurate values of this parameter are required.

## 5. Conclusions

Uniaxial compression tests were conducted on EPS of nominal density *ρ*_nom_ = 20 kg/m^3^ using cubic specimens of 50, 100, 200, and 300 mm side length and rectangular 50 × 50 × 100 mm specimens. Three deformation measurement methods were compared: video extensometer (VE), crosshead displacement at the SUT laboratory (CE-S), and crosshead displacement at the EPS manufacturer’s laboratory (TO-S/TO-S*). The following conclusions were drawn:

- VE measurements provide significantly more accurate determination of EPS elastic parameters in the small-strain range than crosshead displacement-based methods, with VE-based moduli of elasticity up to twice the crosshead displacement values. This has direct implications for both quality control and structural design with EPS.
- The *σ*-*ε*_v_ relationship within *ε*_v_ ≤ 1% is clearly nonlinear and well approximated by a bilinear model. The linear elastic assumption prevalent in the earlier literature is shown to result from the limitations of crosshead displacement measurements rather than true material behaviour.
- Local VE measurements effectively eliminate the size effect and bedding error that significantly affect crosshead displacement results. For global measurements, specimen heights of at least 100 mm are recommended.
- Poisson’s ratio in the small-strain range exhibits a clear dependence on specimen geometry, warranting further investigation given its importance in structural EPS applications.

## Figures and Tables

**Table 1:** Dimensions and number of specimens tested in each laboratory.

Test Location	Specimen Dimensions (mm)	Number of Specimens
Faculty of Civil Engineering laboratory, SUT (groups DIC and CE-S)	50 × 50 × 50	6
	100 × 100 × 100	6
	200 × 200 × 200	6
	300 × 300 × 300	6
	50 × 50 × 100	6
EPS manufacturer’s laboratory (groups TO-S and TO-S*)	50 × 50 × 50	18
	100 × 100 × 100	17 ^1^

^1^ One of the 18 specimens intended for testing was damaged prior to the test.

**Table 2:** Comparison of stress values determined from vertical strain measurements using different gauge lengths.

Specimen Dimensions (mm)	*σ*_A’_/*σ*_B’_ or (*σ*_A’_/*σ*_C’_)		*σ*_A’’_/*σ*_B’’_ or (*σ*_A’’_/*σ*_C’’_)	
	Mean	*CV*	Mean	*CV*
50	1.02	12%	1.04	10%
100	1.04	3%	1.03	2%
200	1.01	2%	1.00	2%
300	1.01	1%	1.00	2%
50 × 50 × 100	1.02	4%	1.01	2%
	(1.03)	(6%)	(1.02)	(3%)

**Table 3:** Comparison of horizontal strains recorded using gauge lengths C and D.

Specimen Dimensions (mm)	*ε*_C_/*ε*_D_			
	For *ε*_v_ = 0.5%		For *ε*_v_ = 1%	
	Mean	*CV*	Mean	*CV*
50	1.10	13%	1.06	4%
100	1.03	4%	0.97	3%
200	1.01	1%	1.00	1%
300	1.00	1%	1.00	2%

## Data Availability

The original contributions presented in this study are included in the article. Further inquiries can be directed to the corresponding author.
